# Proteinase K-pretreated ConA-based ELISA assay: a novel urine LAM detection strategy for TB diagnosis

**DOI:** 10.3389/fmicb.2023.1236599

**Published:** 2023-08-25

**Authors:** Huan Huang, Rong Qu, Kang Wu, Jinchuan Xu, Jianhui Li, Shuihua Lu, Guodong Sui, Xiao-Yong Fan

**Affiliations:** ^1^Shanghai Key Laboratory of Atmospheric Particle Pollution Prevention (LAP3), Department of Environmental Science and Engineering, Fudan University, Shanghai, China; ^2^Shanghai Public Health Clinical Center, Fudan University, Shanghai, China; ^3^School of Laboratory Medicine and Life Science, Wenzhou Medical University, Wenzhou, China; ^4^National Clinical Research Center for Infectious Disease, Shenzhen Third People's Hospital, Shenzhen, China; ^5^Shanghai Institute of Infectious Disease and Biosecurity, Fudan University, Shanghai, China

**Keywords:** *Mycobacterium tuberculosis*, LAM, ConA, ELISA, urine, proteinase K

## Abstract

**Objectives:**

Lipoarabinomannan (LAM), an abundant cell wall glycolipid of mycobacteria including *Mycobacterium tuberculosis* (*Mtb*), is a promising TB diagnostic marker. The current commercially available urine LAM assays are not sufficiently sensitive, and more novel detection strategies are urgently needed to fill the current diagnostic gap.

**Methods:**

A proteinase K-pretreated Concanavalin A (ConA)-based ELISA assay was developed. Diagnostic performance was assessed by several bacterial strains and clinical urine samples.

**Results:**

The limit of detection (LoD) of the assay against ManLAM was 6 ng/ml. The assay reacted strongly to *Mtb* H37Rv and *M. bovis* BCG, intermediately to *M. smegmatis* mc^2^155, and weakly to four non-mycobacteria pathogens. This method could distinguish TB patients from healthy controls (HCs) and close contacts (CCs) in 71 urine samples treated with proteinase K, which increases urine LAM antibody reactiveness. In TB^+^HIV^+^ and TB^+^HIV^−^ patients, the sensitivity was 43.8 and 37.5%, respectively, while the specificity was 100.0%. The areas under ROC curves (AUCs) were 0.74 and 0.82, respectively.

**Conclusion:**

This study implies that ConA can be paired with antibodies to detect LAM. Proteinase K treatment could effectively enhance the sensitivity by restoring the reactiveness of antibodies to LAM.

## Introduction

Tuberculosis (TB), a lung disease caused by *Mycobacterium tuberculosis (Mtb)*, is a leading cause of illness and death worldwide (WHO, 2022). *Mtb* infects 25% of the world's population, and 5–10% may develop TB (WHO, 2022). The COVID-19 pandemic continues to hinder TB diagnosis, resulting in a global decline in new TB diagnoses from 7.1 million in 2019 to 6.4 million in 2021 (WHO, 2022). It is estimated that approximately 40% of new infections go undiagnosed (WHO, 2022), making it more challenging to eradicate TB.

Currently, there are many limitations to TB diagnosis as follows: (1) Bacteriological detection by acid-fast staining and sputum culture is widely used. However, these tests are low-sensitivity or lengthy, respectively (Acharya, 2020). (2) Antigen-specific immunological-mediated detection using tuberculin skin testing (TST) or interferon-gamma release assay (IGRA) is the main method to diagnose *Mtb* infection, but both are unable to differentiate latent TB infection (LTBI) from TB (Acharya, 2020). (3) Nucleic acid amplification tests (NAATs), e.g., Xpert MTB/RIF, are fast and sensitive, but they are not widely available in low- and middle-income countries due to cost (Nadjib, 2022). More innovative diagnostic strategies are needed to overcome these limitations and reduce TB underdiagnosis.

Urine lipoarabinomannan (LAM) could be a promising TB diagnostic biomarker (Flores and Cancino, 2021). LAM, an abundant (approximately 15% of bacterial dry weight) glycolipid located on the mycobacterial cell wall, is a critical virulence component in the *Mtb* infection pathway (Fukuda, 2013). Different LAM derivatives are identified in various groups of mycobacteria, of which ManLAM is a type of LAM with non-reducing mannose caps and is found only in slow-growing mycobacteria (e.g., *Mtb*) (Chatterjee et al., 1992). ManLAM is a glycolipid made up of a mannosyl-phosphatidyl-myo-inositol anchor (MPI), a polysaccharide backbone composed of D-mannan and D-arabinan, and various mannose-capping motifs (Flores and Cancino, 2021). LAM is actively secreted by infected phagocytes or membrane vesicles and may be present in blood, as well as in urine via glomeruli (Prados-Rosales et al., 2011; De, 2015). The amounts of LAM are approximately 1–1,000 ng/ml in sputum (Kawasaki et al., 2019), 2.3–132 pg/ml in serum (Brock et al., 2020), and tens of fg/ml (Chen et al., 2023) to hundreds of ng/ml in urine (Flores and Cancino, 2021). The amounts of LAM in clinical samples collectively reflect the bacterial load, metabolic activity, and degradation ratio of the bacteria in the host body, which mirror the infection status and the responsiveness of anti-TB treatment (Hamasur et al., 2015; Bjerrum et al., 2019; Wood et al., 2019).

There are currently two commercial assays available for LAM-based TB diagnosis: Clearview TB ELISA and Alere DetermineTM TB LAM Ag test (AlereLAM). Clearview TB ELISA is the first one utilizing polyclonal rabbit antibodies (Hamasur et al., 2001), resulting in limited sensitivity and specificity in TB^+^HIV^+^ patients (51 and 94%, respectively), especially in TB^+^HIV^−^ patients (14 and 97%, respectively) (Correia-Neves et al., 2019). AlereLAM is the second one based on colloidal gold immunochromatography assay (ICA) and uses the same polyclonal rabbit antibodies as used in Clearview TB ELISA (Correia-Neves et al., 2019). The result of AlereLAM can be obtained within 25 min using only a 60 μl urine sample, but the sensitivity is also low (42% under 95% specificity in TB^+^HIV^+^ patients). AlereLAM is not suitable for TB^+^HIV^−^ patients due to its low sensitivity (Shah et al., 2016; Bjerrum et al., 2019). Fujifilm SILVAMP TB LAM (FujiLAM) (Fujifilm, Japan), being tested clinically, is an improved LAM-based ICA (70.4% sensitivity and 90.8% specificity in TB^+^HIV^+^ patients) utilizing a pair of high-affinity LAM-specific monoclonal antibodies and a silver amplification step (Mitamura et al., 2013; Broger et al., 2019). However, relatively complex methodology and subjective naked-eye reading induce high error rates and substantial variability (Mitamura et al., 2013; Székely et al., 2022), which hinders its clinical transformation.

Concanavalin A (ConA), a lectin originally extracted from *Canavalia ensiformis* (jack bean), could recognize specifically some internal and non-reducing terminal α-mannosyl and α-glucosyl groups of various sugars or sugar moieties (e.g., LAM, which contains various α-mannosyl groups) (Ij, 1986). Pathogens or their antigens are readily enriched by ConA by binding to their sugar moieties and subsequently detected using a specific antibody. This ConA-antibody strategy can be used for the detection of norovirus (Hong et al., 2015), breast cancer (Choi et al., 2018), and invasive aspergillosis (Raval et al., 2019). CS35 (BEI Resources, USA) is a LAM-specific monoclonal antibody that recognizes the arabinose group, which is different from that recognized by ConA (α-mannosyl group) (Sigal et al., 2018). In theory, the pairing between them could form a stable sandwich structure with LAM. In this study, we first investigated whether ConA and CS35 could be paired to detect LAM in urine. We then developed a simple ELISA assay with proteinase K pretreatment and tested its performance in lysates of several bacterial strains and clinical urine samples for TB diagnosis.

## Materials and methods

### Materials and reagents

CS35 and HRP-goat anti-mouse IgG antibodies were purchased from BEI Resources (USA) and Biodragon (China), respectively. ConA and BSA were obtained from Sigma–Aldrich (USA). Middlebrook 7H9 broth and oleic acid-albumin-dextrose-catalase enrichment (OADC) were purchased from BD Difco (USA). The vacuum freeze dryer was purchased from Labconco (USA). HiPrep 16/60 Sephacryl S-100 HR column was obtained from Cytiva (USA). Moreover, the 96-well plates were purchased from Greiner (Germany). 3,3',5,5'-Tetramethylbenzidine (TMB) substrate was obtained from Millipore (USA) substrate. Alexa Fluor® 594-conjugated ConA was obtained from Invitrogen (USA). All other chemicals were of analytical purity. *Mycobacterium tuberculosis* H37Rv (ATCC 27294), *Mycolicibacterium smegmatis* (ATCC 700084), and *Mycobacterium bovis* BCG (ATCC 35734) were preserved in our laboratory. *Klebsiella pneumoniae* (ATCC 10031), *Pseudomonas aeruginosa* (ATCC 27853), and *Acinetobacter Baumannii* (ATCC 19606) were provided by Jianhui Li (Shanghai Public Health Clinical Center). *E. coli* Top10 was purchased from Sangon Biotech (China).

### Urine sample collection

The Ethics Committee of Shanghai Public Health Clinical Center (SPHCC) approved this study (No. 2022-S044-03), and all patients enrolled in this study signed a written informed consent form. From November 2018 to April 2019, 71 urine samples were collected from 13 healthy controls (HCs), 7 close contacts (CCs) of TB patients, 48 bacteriologically confirmed tuberculosis, and 3 non-tuberculosis mycobacteria (NTM)-infected patients (Table 1). Each patient was required to provide mid-stream urine samples, which were kept at 4°C within 2 h and then immediately moved to −80°C until analysis. All CCs were without TB symptoms, or abnormal CXR, but shared the same enclosed living space for at least 1 night/week or extended periods during the day with the active tuberculosis (ATB) patient for 3 months before the diagnosis of TB. All HCs were without TB symptoms, abnormal CXR, and any contact with a pulmonary tuberculosis patient. All TB patients enrolled in this study had at least one TB-specific positive assay of culture, smear, and Xpert MTB/RIF. Of them, 32 were HIV-negative (10 for extrapulmonary TB and 22 for pulmonary TB), and 16 were HIV-positive. All participants were negative for hepatitis B virus (HBV) and HCV.

**Table 1:** Demographic and clinical characteristics.

	**HCs (*n =* 13)**	**CCs (*n =* 7)**	**EPTB (*n =* 10)**	**PTB (*n =* 22)**	**TB^+^HIV^+^ (*n =* 16)**	**NTM (*n =* 3)**	***p***
**Age, years**	26 (25–32)	41 (32–50)	27 (17–47)	51.5 (27–65)	35.5 (31–63)	61 (11–64)	>0.05
**Sex**							>0.05
Women	6 (46%)	3 (43%)	3 (30%)	7 (32%)	3 (19%)	0	
Men	7 (54%)	4 (57%)	7 (70%)	15 (68%)	13 (81%)	3 (100%)	
**Bacterial test**							<0.05
Culture+ and/or Smear+			10 (100%)	16 (73%)	7 (44%)	3 (100%)	
Culture– and Smear–			0	6 (27%)	9 (56%)	0	
**T-SPOT**.***TB***							<0.05
+		0	9 (90%)	21 (95%)	7 (44%)	2 (67%)	
-		7 (100%)	0	1 (5%)	9 (56%)	1 (33%)	
**Xpert MTB/RIF**							<0.05
+			9 (90%)	14 (64%)	4 (25%)	0	
–			1 (10%)	8 (36%)	2 (13%)	3 (100%)	
**CD4 count, cells/μl**					65 (24–112)		NA
**Data are median (IQR) or** ***n*** **(%)**							

### Bacterial strains used and their culture conditions

*Mycobacteria* strains were grown at 37°C in liquid Middlebrook 7H9 broth supplemented with 10% (v/v) OADC, 0.5% glycerol, and 0.05% Tween-80. *Klebsiella pneumonia, Pseudomonas aeruginosa, Acinetobacter Baumannii*, and *E. coli* Top10 were grown in a liquid LB medium. The cultures in the exponential stage were frozen at −80°C as stock after adding glycerol (final concentration 10%).

### Purification, identification, and quantification of ManLAM

ManLAM was purified from a clinical isolate of *Mtb* following the procedures described previously (Torrelles et al., 2006; Grzegorzewicz and Jackson, 2013; Pan et al., 2014). In brief, the *Mtb* pellet was inactivated at 80°C for 30 min. The bacteria were resuspended in chloroform/methanol (2:1, v/v) and then placed in a shaking incubator at 37°C for 12 h. Then, the collected pellet was resuspended in chloroform/methanol/water (10:10:3, v/v/v) solution and placed in a shaking incubator at 37°C for 12 h. The collected pellet was freeze-dried overnight using a vacuum freeze dryer. The dried pellet was resuspended in PBS (containing DNase, RNase, lysozyme, and PMSF) and lysed via ultrasonication. The lysed sample was placed at 37°C for 2 h. Then, Triton X-114 was added (final concentration 8%) and incubated overnight at 4°C. The solution was centrifuged at 27,000 *g* for 1 h, and the supernatant was collected and placed at 37°C until obvious stratification. The lower organic phase was collected, added with anhydrous ethanol, and incubated overnight at −20°C. The pellet was collected via centrifugation and freeze-dried. The pellet was resuspended in PBS containing proteinase K (final concentration: 2 mg/ml), incubated in a water bath at 37°C for 2 h, and then dialyzed against PBS at 4°C. The dialyzed solution was freeze-dried. The crude LAM powder was resuspended in PBS and applied to a HiPrep 16/60 Sephacryl S-100 HR column. Fractions of effluent were collected.

Fractions of effluent containing ManLAM were identified via glycogen staining (Tang et al., 2016). In brief, after separating the fractions of effluent in sodium dodecyl sulfate-polyacrylamide gel electrophoresis (SDS-PAGE) gel, ManLAM contained in the gel was oxidized with periodate to form an aldehyde group, followed by staining with Schiff reagent. The Schiff reagent was prepared from basic fuchsin and sodium sulfite, which reacts with aldehyde groups to form an amaranth substance. ManLAM-positive fractions of effluent were pooled, dialyzed against PBS, and quantified via the orcinol sulfuric acid method as described previously (Yin et al., 2013). The residual protein content in the dialyzed/pooled ManLAM sample was also routinely quantified via the BCA method. The dialyzed/pooled ManLAM sample was re-validated via glycogen staining and Western blotting using biotin-labeled ConA/HRP-Streptavidin.

### ConA/CS35-based ELISA for ManLAM detection

“ConA/CS35” in this study means that ConA was tested as a capture protein and CS35 was tested as the detection antibody. ConA was diluted with PBS containing 0.1 mM CaCl_2_ (PBSCa). ELISA plate wells coated with (serially diluted) ConA (100 μl/well) were incubated at 4°C overnight. The next morning, the plate wells were blocked with 1% BSA, and then the samples to be tested were added (ManLAM in PBS or urine, bacterial strain lysis, and clinical samples) and incubated for 1 h at room temperature. Negative wells were incubated with PBS. After washing with PBS containing 0.05% tween-20 (PBST) five times, the plate wells were added with CS35 (100 μl/well, final concentration: 2 μg/ml, dissolved in PBS) and incubated for 1 h at room temperature. After washing with PBST five times, the plate wells were added with 100 μl HRP-goat anti-mouse IgG (100 μl/well, diluted 1:5000 in PBS) and incubated for 30 min at room temperature. The plate wells were rinsed six times with PBST, then added with TMB substrate (100 μl/well), and incubated at room temperature for 10 min. The reaction was terminated by adding 2 M H_2_SO_4_ (50 μl/well), and OD_450_ was measured. CS35 was also tested as a capture antibody, and accordingly, ConA was tested as a detection protein (i.e., CS35/ConA-based ELISA). Alexa Fluor® 594-conjugated Con was also diluted with PBS and used to visualize the dose-dependent coating pattern in ELISA plate wells under a fluorescent microscope.

### The limit of detection (LoD) of ConA/CS35-based ELISA to ManLAM and the reactiveness of ConA/CS35-based ELISA to bacterial strains

ELISA plate wells were coated with ConA (200 μg/ml) (100 μl/well). For LoD analysis, serial diluted ManLAM samples were used. For analyzing the reactiveness of ConA/CS35-based ELISA to other strains, several different strains were used including two *Mtb* complex (MTBC) strains (i.e., *Mtb* H37Rv and *M. bovis* BCG Pasteur), one non-tuberculous mycobacterium (NTM) (i.e., *M. smegmatis* mc^2^155), and four non-mycobacteria strains (i.e., *Klebsiella pneumoniae, Pseudomonas aeruginosa, Acinetobacter baumannii*, and *E. coli* Top10). The fresh cultures of the strains were adjusted to OD_600_ = 1 and inactivated at 80°C for 30 min. The pellets from 1 ml liquid were washed twice with PBS, resuspended with 1 ml PBS, and lysed via ultrasonication. The diluted lysed liquids (1:100 with PBS) were added into plate wells (100 μl/well) for comparative analysis.

### The treatment of urine samples with proteinase K

The urine sample (300 μl) was treated with proteinase K (final concentration 200 μg/ml) at 37°C for 30 min and then inactivated at 100 °C for 3 min (Shah et al., 2016). The supernatants were collected via centrifugation (12,000 × *g*, 10 min) for ConA/CS35-based ELISA.

### Statistical analysis

Statistical analysis was performed using SPSS (version 27), and the data were visualized using GraphPad Prism software (version 8.0.1). The Shapiro–Wilk test was used to determine the normality of the data. Unpaired, two-tailed Student's *t*-test was used to access the statistical significance between the two groups. One-way ANOVA with Tukey's tests or Games–Howell's multiple comparisons tests were used to access the statistical significance among multiple experimental groups. The 95% confidence interval (CI) of the ROC curve was calculated using the method of Wilson/Brown.

## Results

### ConA/CS35-based ELISA could detect ManLAM

**Figure 1:**
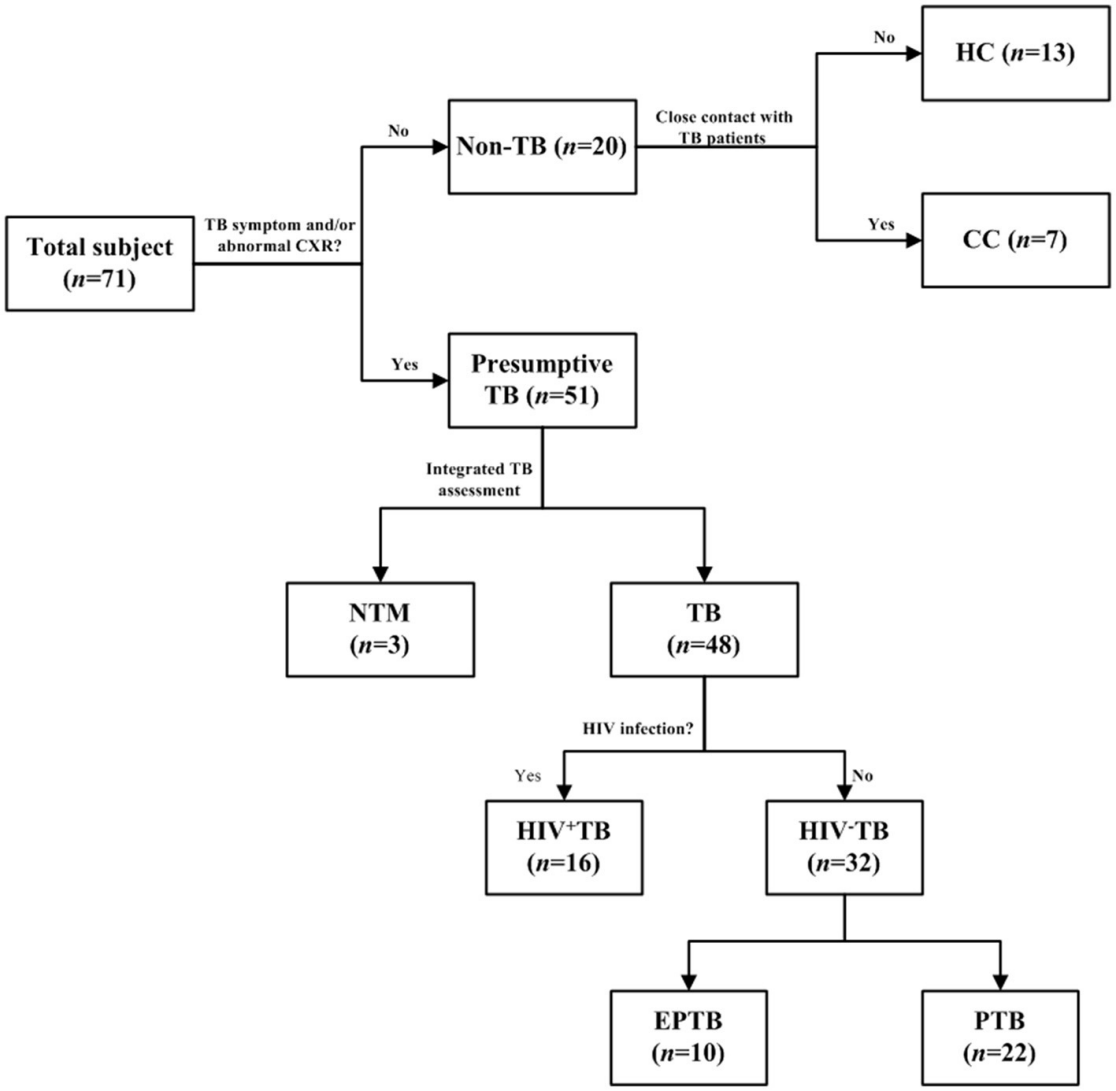
Selection flow of recruited patients. The ConA/CS35 was evaluated with cryopreserved urine samples from individuals with tuberculosis, NTM infection, and non-tuberculosis. CC, close contact: no TB symptom and abnormal CXR and close contact with TB patient 2 weeks before recruitment. HC, health control: no TB symptom and abnormal CXR and no history of exposure to TB. TB, tuberculosis: the diagnosis of a TB patient was confirmed by one or a combination of smear, culture, and GeneXpert. NTM, non-tuberculous mycobacteria; EPTB, extrapulmonary tuberculosis; PTB, pulmonary tuberculosis.

As shown in Figure 1, the molecular weight of ManLAM purified from a clinical isolate of *Mtb* is between 35 and 45 kDa, which is consistent with ManLAM extracted from other *Mtb* strains (Torrelles et al., 2006; Choudhary et al., 2018). The purified ManLAM was used for subsequent analysis.

**Figure 2:**
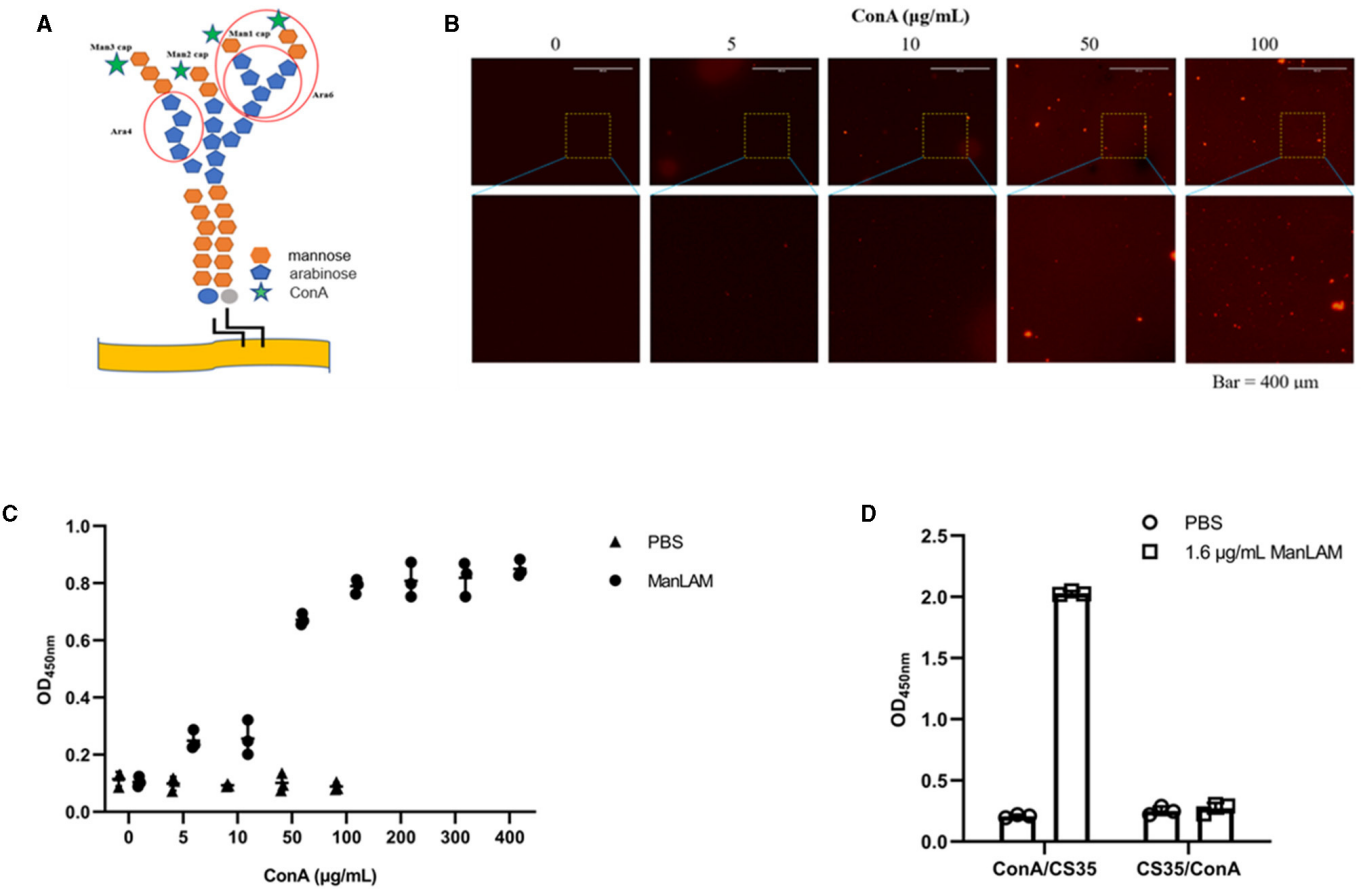
ConA/CS35-based ELISA could detect ManLAM. **(A)** The illustration of epitopes recognized by ConA and CS35. The red circles present the recognized epitopes of CS35, which mainly recognize tetra-arabinoside and branched hexa-arabinoside (Mitamura et al., 2013). Green star: ConA, which recognizes some mannosyl groups (Correia-Neves et al., 2019). **(B)** The dose-dependent coating of Alexa Fluor 594-conjugated ConA on ELISA plate. **(C)** The ELISA detection of ManLAM (10 μg/ml of ManLAM was used, and the LAM solution and ConA coated were reacted at room temperature for 1.5 h.), of which the ELISA plate wells were coated with serial diluted ConA. CS35 was used as the detection antibody. **(D)** The comparative detection of ManLAM between ConA/CS35-based ELISA and CS35/ConA-based ELISA. “ConA/CS35” means that the capture protein was ConA and the detection antibody was CS35, whereas “CS35/ConA” means that the capture antibody was CS35 and the detection protein was ConA. Data are means ± SD of three or four biological repeats. ^****^P < 0.0001 (unpaired, two-tailed Student's *t*-test).

Figure 2A shows that the epitopes of ManLAM that ConA and CS35 recognize are different. ConA recognizes the α-mannosyl groups of ManLAM, whereas CS35 binds to the arabinosyl groups of LAM (Sigal et al., 2018). Moreover, we tested whether ConA could be paired with CS35 to detect ManLAM in the ELISA platform. We observed that ConA could be coated to ELISA plate wells in a dose-dependent manner (Figure 2B), which resulted in the dose-dependent capturing of ManLAM, and the signals plateaued at 200 μg/ml (i.e., 20 μg in 100 μl) when using CS35 as detection antibody (Figure 2C). In contrast to ConA/CS35-based ELISA, CS35/ConA-based ELISA (i.e., CS35 being used as a capture antibody and ConA being used as detection protein) could not detect ManLAM (Figure 2D). In conclusion, ConA/CS35-based ELISA could successfully detect ManLAM.

### The LoD of ConA/CS35-based ELISA to ManLAM

**Figure 3:**
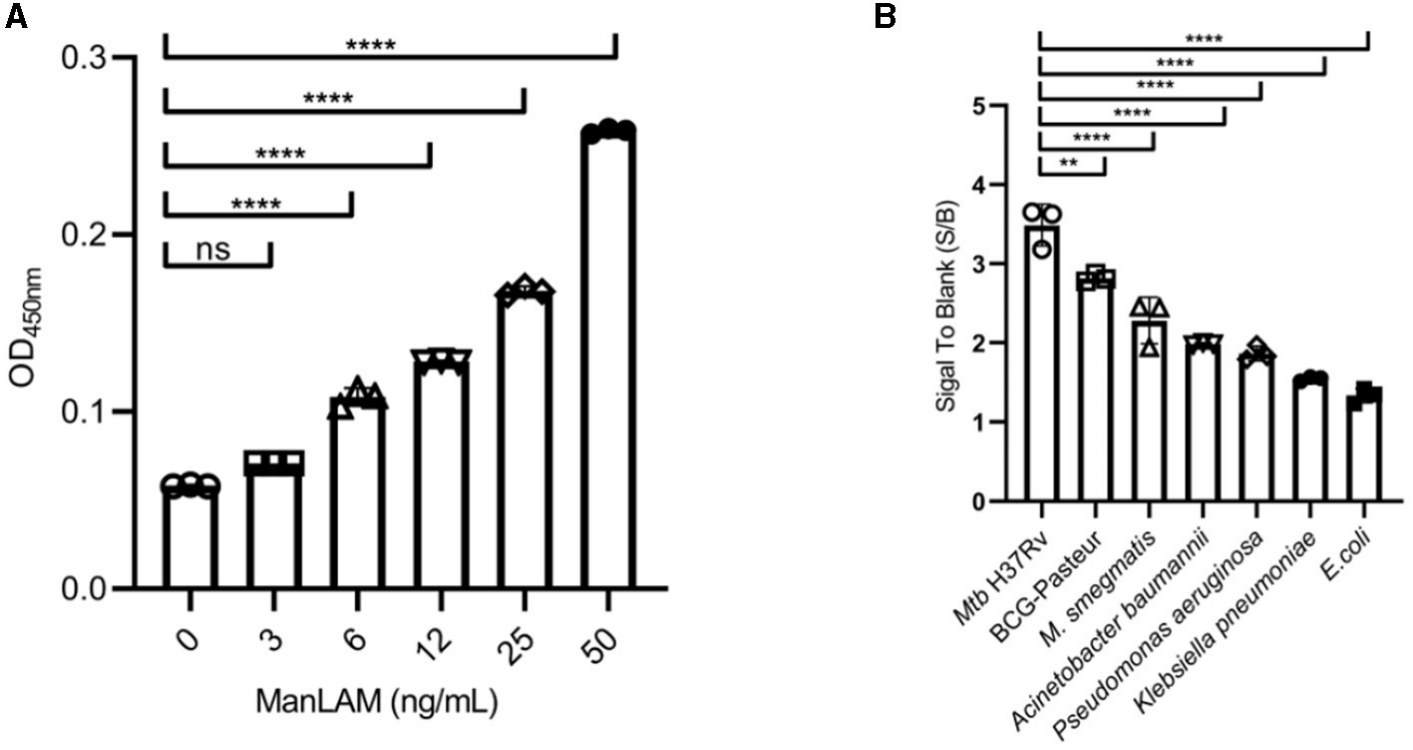
Diagnostic performance of the ConA/CS35-based ELISA to **(A)** ManLAM standards and **(B)** several strains of bacteria. ManLAM standards and bacterial lysates were diluted through PBS buffer and did not pretreat via proteinase K. Data are means ± SD of three or four biological repeats. ^ns^*P* > 0.05, ^**^P < 0.01, ^****^P < 0.0001 (one-way ANOVA with Tukey's multiple comparison tests).

As shown in Figure 3A, the LoD of ConA/CS35-based ELISA to ManLAM was 6 ng/ml when coating ConA at 200 μg/ml (i.e., 20 μg in 100 μl).

### The reactiveness of ConA/CS35-based ELISA to several other strains

As shown in Figure 3B, the assay displayed the highest signals to two *Mtb* complex strains (i.e., *Mtb* H37Rv and *M. bovis* BCG Pasteur), intermediate signals to one non-tuberculous mycobacteria (i.e., *M. smegmatis* mc^2^155), and the lowest signals (S/B ratio < 2) to four non-mycobacteria pathogens (i.e., *Klebsiella pneumoniae, Pseudomonas aeruginosa, Acinetobacter baumannii*, and *E. coli* Top10).

### The signals in urine samples using ConA/CS35-based ELISA

**Figure 4:**
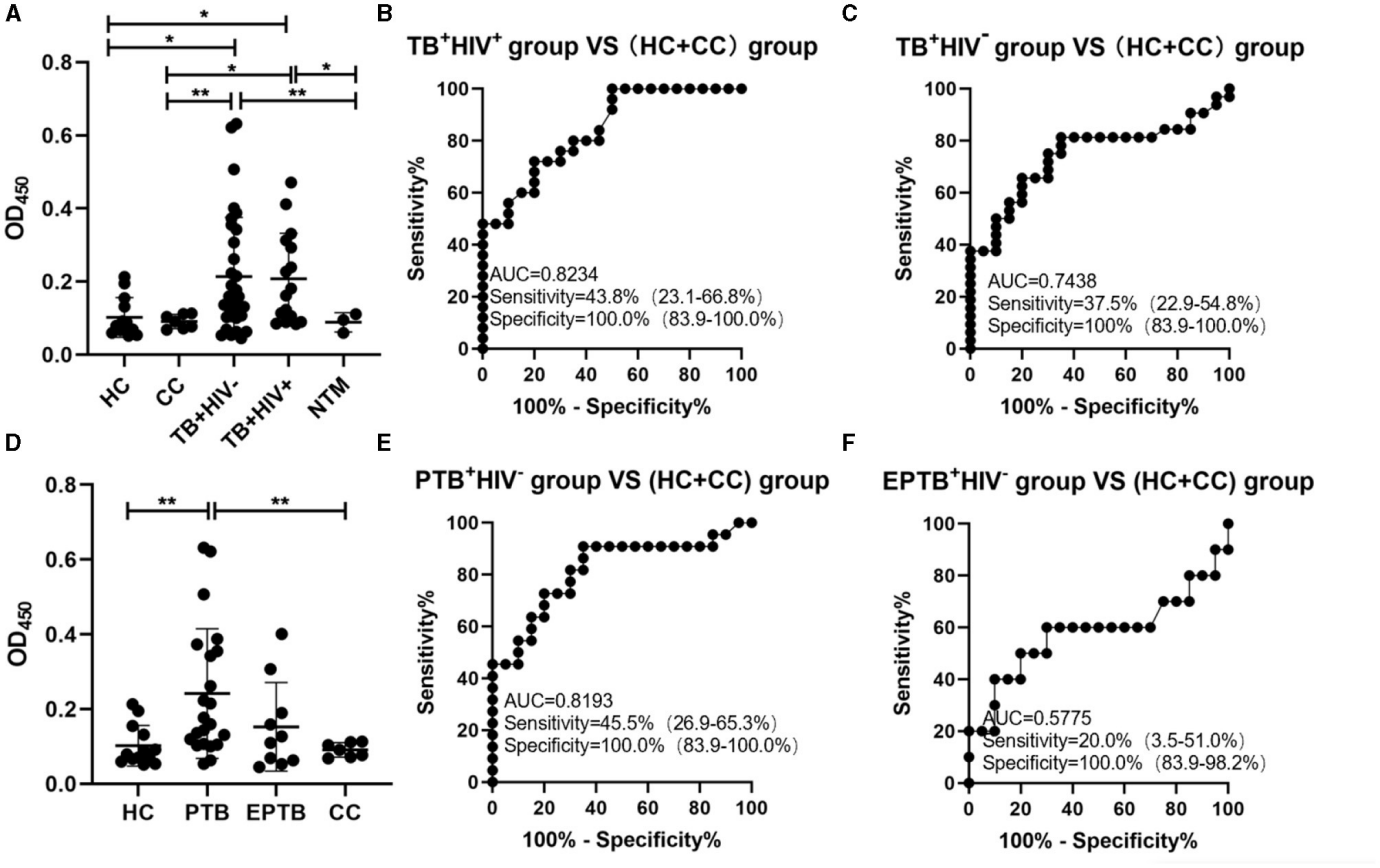
ConA/CS35-based ELISA using urine samples. **(A** and **D)** The signals detected using ConA/CS35-based ELISA were subtracted by the signals of negative wells (i.e., PBS). **(B**, **C**, **E**, and **F)** ROC analysis. ^*^P < 0.05, ^**^P < 0.01 (one-way ANOVA with Games–Howell's multiple comparison tests).

The signals in urine samples of TB patients (either TB^+^HIV^−^ or TB^+^HIV^+^) were higher than the signals in HC, CC, and NTM; there was neither any difference in signals between the HC and CC groups nor between the TB^+^HIV^+^ and TB^+^HIV^−^ groups (Figure 4A). The sensitivities of the assay for TB^+^HIV^+^ patients and TB^+^HIV^−^ patients were 43.8% (95% CI: 23.1–66.8%) and 37.5% (95% CI: 22.9–54.8%), respectively, under the specificity of 100% (95% CI: 83.9–100.0%); the AUC was 0.8234 for TB^+^HIV^+^ patients and 0.7438 for TB^+^HIV^−^ patients (Figures 4B, C). When TB^+^HIV^−^ patients were further divided into PTB and EPTB, the signals were higher in PTB patients than in HCs, but there was no statistical difference between EPTB patients and HCs (Figure 4D). The sensitivities of the assay for PTB^+^HIV^−^ and EPTB^+^HIV^−^ patients were 45.5% (95% CI: 26.9–65.3%) and 20% (95% CI: 3.5–51.0%), respectively, under the specificity of 100.0% (95% CI: 83.9–100.0%); the AUC was 0.8193 for PTB^+^HIV^−^ patients and 0.5775 for EPTB^+^HIV^−^ patients (Figures 4E, F).

## Discussion

The only urine LAM assay approved by the World Health Organization is AlereLAM, but its clinical application may be limited for its low sensitivity, especially in those without HIV. Thus, the detection of urinary LAM requires additional innovative assays to fill the current diagnostic gap, such as new signal amplification techniques (e.g., FujiLAM), high-affinity and specific antibody screening, and non-antibody-based assays (e.g., aptamer and ConA). In this study, we developed a simple ConA/CS35-based ELISA assay for urine LAM detection, based on the principle that ConA and the monoclonal antibody CS35 can recognize different LAM epitopes (Choudhary et al., 2018; WHO, 2022). To the best of our knowledge, this is the first research endeavor to utilize non-antibody protein for the detection of LAM. ConA is much cheaper than antibodies (approximately 50 times) (Hong et al., 2015); therefore, the ConA-antibody paired assays have the potential to lower the cost of TB testing, especially with the development of ConA-based point-of-care test, which will be beneficial for TB diagnosis in resource-limited areas.

The sensitivity of urine LAM analysis seems to be positively correlated with the LoD of the method. In a study addressing TB^+^HIV^−^ patients, AlereLAM, FujiLAM, and EclLAM had sensitivities of 10.8, 53.2, and 66.7%, respectively, and EclLAM and FujiLAM had LoDs of 5 and 10–20 pg/ml, respectively, which were at least 10 times lower than AlereLAM (Broger et al., 2020). In our study, the LoDs of ConA/CS35 ELISA were only 6 ng/ml; however, the sensitivity in HIV-negative patients was 37.5% (higher than Alere) when the specificity was 100%. This seems to be explained by the formation of complexes between LAM and proteins in the urine sample. These complexes may spatially affect the correct binding/reactivity of the antibody to LAM and can be largely restored by proteinase K treatment in our ConA/CS35 ELISA assays and capture ELISA (Amin et al., 2018). It is suggested that proteinase K treatment is a successful sensitivity booster for urine LAM detections.

The specificity of urine LAM analysis depends on the reactivity of the ligands used (e.g., antibodies) between different bacterial strains. The LAM-specific polyclonal antibodies utilized in Clearview TB ELISA and AlereLAM could react to mycobacteria stains from both MTBC and NTM strains and could not react to the tested non-mycobacteria pathogens (Amin et al., 2018). The monoclonal antibody, being used as a gold particle-conjugated antibody in FujiLAM or a capture antibody in EclLAM, reacts strongly to MTBC strains and reacts weakly or could not react to the tested NTM strains and non-mycobacteria pathogens (Sigal et al., 2018). ConA could react to some mannosyl group-containing sugars (Ij, 1986), which are non-specifically present in various bacteria or viruses. However, CS35, a relatively specific monoclonal antibody that recognizes mostly MTBC and few NTM strains (Boehme et al., 2005), can compensate for the lack of ConA's specificity. As a result, ConA/CS35-based ELISA could react strongly to *Mtb* H37Rv and *M. bovis* BCG but react weakly to other non-mycobacteria pathogens (Figure 3B).

However, this study has the following limitations. First, even with proteinase K pretreatment, the sensitivities of ConA/CS35 ELISA in urine from HIV TB patients were 37.5% and 43.8%, which is similar to AlereLAM [almost 42% in HIV-positive adults (Bjerrum et al., 2019)] but lower than Fuji LAM [75% in HIV-positive (Li et al., 2021)]. However, it should be noted that this is only a preliminary study demonstrating the availability of ConA paired with monoclonal antibodies, and the enhanced effect of Proteinase K treatment on sensitivity was achieved. Further improvements in this method, such as the replacement of higher affinity antibodies or stronger signal probes (e.g., electrochemiluminescence and quantum dots), are very likely to improve the current diagnostic performance. Second, as a method of testing non-sputum samples, our test duration is still too long (~4 h), which is longer than AlerelAM (25 min) and FujiLAM (60 min) (Bulterys et al., 2019). However, we are certain that this assay may readily be converted to a POCT version (e.g., test strips) with appropriate improvements. Simultaneous improvements in the sensitivity and test time of ConA-based LAM assays could further enhance the attractiveness of this method as a cost-effective and easy-to-use TB screening tool, especially in low-resource communities with a higher TB burden. Third, we did not evaluate the effect of the use or no use of proteinase K on the improvement of sensitivity in this study. However, the enhancement of LAM analysis by proteinase K has been confirmed by several studies (Amin et al., 2021; Clarke et al., 2023; Panraksa et al., 2021), based on a different LAM analysis than ours. The role of proteinase K in the ConA/CS35 ELISA will be further elucidated in our further studies.

In conclusion, we established a simple ELISA assay for urine LAM detection for TB diagnosis. Our assay demonstrates that ConA can be paired with antibodies to detect LAM in urine, broadening the idea of non-antibody-based methods. In addition, our results show that proteinase K treatment could effectively enhance sensitivity by restoring the reactiveness of antibodies to LAM.

## Data availability statement

The original contributions presented in the study are included in the article/Supplementary material, further inquiries can be directed to the corresponding authors.

## Ethics statement

The studies involving humans were approved by the Shanghai Public Health Clinical Center (SPHCC) Ethics Committee. The studies were conducted in accordance with the local legislation and institutional requirements. The participants provided their written informed consent to participate in this study.

## Author contributions

HH, RQ, KW, and X-YF conceived and designed the experiments and wrote the manuscript. HH, RQ, and KW acquired, analyzed, and interpreted the data. GS, KW, and X-YF contributed to overall supervision, critical comments, and the manuscript review. GS, JL, JX, and SL gave critical comments on the manuscript. All authors read and approved the final manuscript.

## Supplementary material

Figure S1 The identification of purified ManLAM. 2 μg of ManLAM was added to each lane for electrophoresis, and ManLAM on SDS gel was oxidized by periodate followed by Schiff reagent staining **(left)** or biotin-labeled ConA recognition followed by visualized reaction of HRP-Streptavidin **(right)**. M, protein marker. kD, kiloDalton.
