# Supplementary figures and images for: Proteinase K-pretreated ConA-based ELISA assay: a novel urine LAM detection strategy for TB diagnosis
Source: Front Microbiol. 2023 Aug 25;14:1236599. doi: 10.3389/fmicb.2023.1236599 (PMC10485274; doi:10.3389/fmicb.2023.1236599)

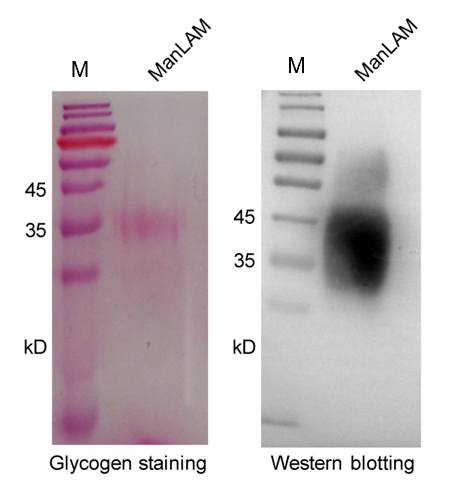

Supplement: Figure S1 — The identification of purified ManLAM. 2 μg of ManLAM was added to each lane for electrophoresis, and ManLAM on SDS gel was oxidized by periodate followed by Schiff reagent staining (left) or biotin-labeled ConA recognition followed by visualized reaction of HRP-Streptavidin (right). M, protein marker. kD, kiloDalton. [file Image_1.tif]
